# Clinical Features and Outcome in Children with Severe *Plasmodium falciparum* Malaria: A Meta-Analysis

**DOI:** 10.1371/journal.pone.0086737

**Published:** 2014-02-06

**Authors:** Laurens Manning, Moses Laman, Wendy A. Davis, Timothy M. E. Davis

**Affiliations:** School of Medicine and Pharmacology, University of Western Australia, Fremantle Hospital, Fremantle, Western Australia, Australia; University of Barcelona, Spain

## Abstract

**Background:**

Although global malaria mortality is declining, estimates may not reflect better inpatient management of severe malaria (SM) where reported case fatality rates (CFRs) vary from 1–25%.

**Methods:**

A meta-analysis of prospective studies of SM was conducted to examine i) whether hypothesized differences between clinical features and outcome in Melanesian compared with African or Asian children really exist, and ii) to explore temporal changes in overall and complication-specific CFRs. The proportions of different SM complications and, overall and complication-specific CFRs were incorporated into the meta-analysis. Adjustments were made for study-level covariates including geographic region, SM definition, artemisinin treatment, median age of participants and time period.

**Findings:**

Sixty-five studies were included. Substantial heterogeneity (*I* ^2^>80%) was demonstrated for most outcomes. SM definition contributed to between-study heterogeneity in proportions of cerebral malaria (CM), metabolic acidosis (MA), severe anemia and overall CFR, whilst geographic region was a significant moderator in for CM and hypoglycemia (HG) rates. Compared with their African counterparts, Melanesian children had lower rates of HG (10% [CI95 7–13%] versus 1% [0–3%], *P*<0.05), lower overall CFR (2% [0–4%] versus 7% [6–9%], *P*<0.05) and lower CM-specific CFR (8% [0–17%] versus 19% [16–21%], *P*<0.05). There was no temporal trend for overall CFR and CM-specific CFR but declining HG- and MA- specific CFRs were observed.

**Interpretation:**

These data highlight that recent estimates of declining global malaria mortality are not replicated by improved outcomes for children hospitalized with SM. Significant geographic differences in the complication rates and subsequent CFRs exist and provide the first robust confirmation of lower CFRs in Melanesian children, perhaps due to less frequent HG.

## Introduction

Although most of the 500 million clinical cases of malaria due *Plasmodium falciparum* estimated to occur globally are uncomplicated [1], some (1–4%) are considered severe enough to require hospitalization and/or parenteral antimalarial treatment [2]. In children, the term ‘severe malaria’ is currently applied when asexual forms of *P. falciparum* are detected in the peripheral blood and there is impaired consciousness or coma, prostration, multiple seizures, hyperlactatemia or metabolic acidosis, severe anemia, dark urine, hypoglycemia, jaundice, respiratory distress, persistent vomiting, abnormal bleeding, shock, and/or renal failure [3]. These clinical criteria reflect the World Health Organization (WHO) 2000 definition of severe malarial illness [3] that is based on descriptive studies of African children and Asian adults with severe *P. falciparum* infections. It has evolved from previous iterations, published in 1986 [4] and 1990 [5], that were developed to identify patients most at risk of death and to provide standard criteria for research studies [3].

Both the proportions of children with different clinical features defining severity and their subsequent case fatality rates (CFRs) will depend on selection/recruitment strategies, the sensitivity of malaria diagnosis/speciation and the definitions of the features themselves, as well as inpatient management including identification of co-incident disease, and social, cultural and genetic factors that could influence presentation and clinical course. Apparent inconsistencies between published studies may reflect differences in one or more such factors. Indeed, reported CFRs in severe childhood malaria studies vary from 1–25% [6], [7], with the suggestion that Melanesian children have a better prognosis than African children [8], [9]. However, there has also been a temporal change in global malaria-associated mortality, with a peak in 2004 and subsequent fall to an estimated 1,238,000 deaths in 2010 [10]. These estimates have been derived from vital registration (VR) of cause of death and verbal autopsy (VA) studies, augmented by estimates of the contribution of malaria to non-specific causes of death such as anemia and fever based on local malaria epidemiology. Case fatality rates (CFRs) of children hospitalized with severe malaria are, however, not incorporated into overall malaria mortality estimates. Because of this, it cannot be inferred that improved inpatient management of such cases has contributed to the recent decline in malaria-attributable mortality [10].

In the light of this background, we performed a standardized review and meta-analysis to examine whether i) there are significant differences between clinical features and outcome in Melanesian children with severe malaria and those in cases from Africa or Asia, and ii) there have been changes in overall and complication-specific CFRs over time. We incorporated geographic region, the year in which the study was performed, the case definition used, patient age and antimalarial therapy as potential moderators of outcome.

## Methods

The supporting PRISMA checklist is available as Checklist S1.

### Search strategy and selection criteria

A search of all descriptive studies of severe childhood falciparum malaria published before the end of 2012 was conducted using Medline. The following MeSH terms were employed: “child*” OR “pediatric” OR “paediatric” AND “severe malaria” OR “cerebral malaria” OR “severe anemia” OR “severe anaemia” OR “severe malarial anemia” OR “severe malarial anaemia” OR “metabolic acidosis” OR “lactic acidosis”. The bibliographies of key papers were reviewed and additional studies obtained. Publications in French were included but those describing children with severe *P. vivax* or mixed *P. vivax/P. falciparum* malaria were not.

The definition of severe malaria was categorized as ‘all malaria requiring admission’, ‘clinician-determined severe malaria’, WHO 1990 [5] and WHO 2000 [3]. The latter two definitions were applied if explicitly stated or if the clinical features were consistent with the published criteria. Unless otherwise stated, included studies were prospective with observational, case-control or interventional designs and with clearly defined clinical features of severity and/or reported CFRs. In antimalarial treatment trials, the proportions of children with specific features of severity in each treatment arm were pooled but CFRs were calculated by allocated treatment with artemisinin derivatives or with quinine/aminoquinoline therapy. Studies were grouped according to whether they were conducted in Africa, Asia or Papua New Guinea (PNG; Melanesia). The median or mean age of children reported for each study was dichotomized as <4 or ≥4 years. Analysis of temporal trends was based on the year the study was conducted, or the median for those running for more than a year, rather than the year of publication.

Studies focusing on children with only severe malarial anemia, cerebral malaria, hypoglycemia and metabolic acidosis were included only in CFR analyses of the relevant specific clinical feature. The term ‘deep coma’, ‘unrousable coma’, ‘comatose not responding to painful stimuli’ were considered synonymous. This equated to a Blantyre Coma Score (BCS) [11] of ≤2 in most instances, but deep coma was defined as a BCS ≤3 in three studies [12]–[14] and, in another [15], the Adelaide coma scale was used. For publications reporting ‘deep coma’, subgroups of children with BCS scores of 0, 1 and 2 were also extracted when available.

Definitions of severe malarial anemia (hemoglobin <50 g/L) and hypoglycemia (blood/plasma glucose <2.2 mmol/L) were uniform across time, geographic region and WHO definition. Either hyperlactatemia (blood/plasma lactate ≥5.0 mmol/L), or serum bicarbonate ≤15 mmol/L or ≤12.2 mmol/L [16] or base excess <−8 mmol/L, were considered indicative of metabolic acidosis. A severe overlap syndrome of deep coma, severe anemia, and metabolic acidosis, hyperlactemia or respiratory distress, in a single child was reported in a number of studies and was, therefore, considered a separate presenting clinical feature.

### Statistical analysis

The R statistical package ‘metafor’ was used for meta-analyses [17]. Briefly, all outcomes of interest were considered proportions for random effects models. Study level covariates or ‘moderators’ including geographic region, the definition of severe malaria used, treatment with an artemisinin derivative, patient age and the year of study were incorporated as appropriate into mixed effects models. If a variable of interest in an individual study had a value of 0 but was measured, a value of 0.01 was assumed to allow its inclusion in the meta-analysis.

Heterogeneity was determined using the *I* ^2^ statistic, a measure of the percentage of total variability due to between-study heterogeneity. Values <50% suggest a lack of significant heterogeneity. The τ^2^ statistic is a measure of total heterogeneity before and after incorporation of moderator variables, allowing the contribution of moderator variables in mixed effects models to be quantified as a relative proportion of the overall between-study heterogeneity. The mixed effects model with the best fit for each outcome of interest was determined by comparison between derived models using backward stepwise testing, incorporating moderators with a statistically significant effect on heterogeneity, and by using Aikake's Information Criterion and analysis of variance testing at each step to identify the most parsimonious model.

The results of random effect models are depicted using forest plots. In each such plot, the study is denoted by author and year of publication with the exception of the Severe Malaria in African Children (SMAC) studies [18] and the important studies comparing artesunate with quinine therapy in Asia and in African children (denoted as AQUAMAT [19] and SEQUAMAT [20], respectively). Publication bias was assessed by visual inspection of funnel plots. For each outcome of interest, funnel plots with (the final mixed effects model) and without (random effects) moderator effects were generated. Meta-regression plots for outcomes of interest according to the year of the study were also generated displaying a meta-regression line and 95% confidence intervals (CI95).

### Registration

This study is registered with PROSPERO (CRD42013005510).

## Results

### Studies included in the meta-analysis

**Figure 1:**
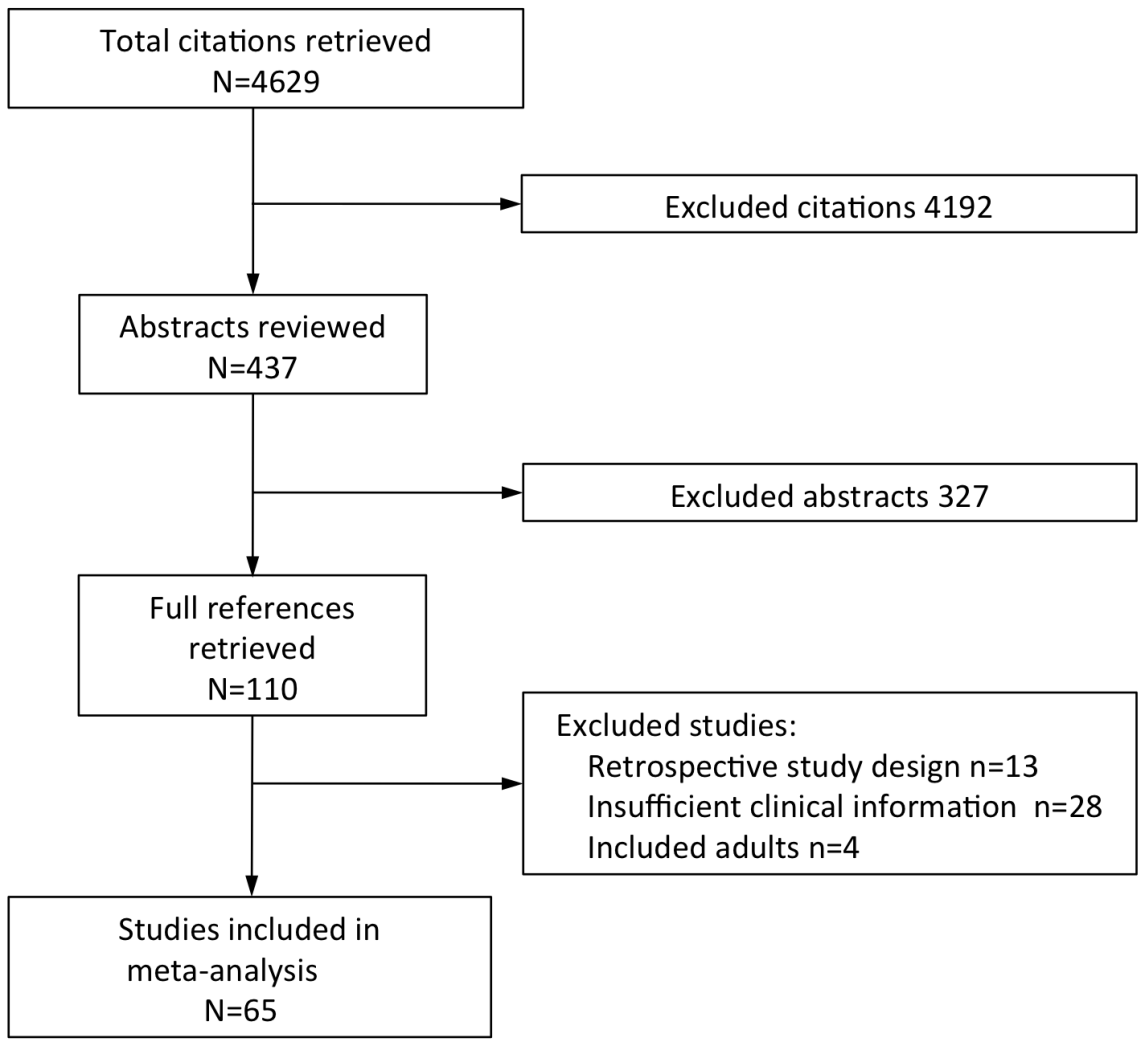
Flow diagram showing studies included in meta-analysis.

The initial Medline search yielded 4,629 citations. A PRISMA [21] flow diagram outlining the determination of which studies were included in the meta-analysis is shown in Figure 1. Sixty-five studies were included (see Table 1) [2],[6],[7],[11]–[15],[19],[20],[22]–[75]. One was reported as a retrospective study, but the data were of good quality and collected prospectively [30]. Most (n = 55) were from Africa, with 6 and 4 studies from Asia and PNG, respectively. The SMAC network of studies contributed 10 data points to the meta-analysis. The data on proportions of different clinical features from the 6 SMAC sites are published in a single paper [73], while additional data from the same study sites are detailed elsewhere [36], [44]. One study included data in two publications from the same cohort [74], [75].

**Table 1:** Description of potential moderator covariates and the number of studies with individual moderators included in meta-analysis.

Moderator of interest		Number of studies (n)
**Region**	Africa	55
	Asia	6
	Melanesia (all from Papua New Guinea)	4
**Definitions of severe malaria**	All malaria admissions	12
	Clinician-determined severe malaria	1
	WHO 1990*	11
	WHO 2000*	18
**Studies describing specific clinical features only**	Cerebral malaria alone	19
	Cerebral malaria and severe anemia	1
**malarieatures of severe malaria only**	Cerebral malaria, shock or hyperparasitemia	1
	Cerebral malaria and hyperlactatemia	1
	Severe anemia alone	1
**Antimalarial medications**	Artemisinin alone	4
	Artemisinin or quinine (comparative study)	16
	Artemisinin or chloroquine	1
	Chloroquine alone	2
	Quinine alone	42

* One study described clinical features for children admitted according to both WHO 1990 and 2000 definitions [12].

The meta-analysis included studies performed from 1987 to 2009 (median 1999) with average patient age ranging from 1 to 8 (median 3) years. Three highly cited articles, all of which reported studies of Melanesian patients, were captured by the search strategy but excluded from analysis. The first, a retrospective study of morbidity surveillance in a cohort of PNG children, did not have detailed data available for ascertainment of clinical manifestations and did not report overall or phenotype-specific CFRs [76]. In the second study, in which all malaria hospitalizations in Irian Jaya, Indonesia were prospectively identified [77], it was not possible to determine the proportions of each clinical manifestation because the group with multiple features was not defined. Furthermore, overall and phenotype-specific CFRs were not reported according to age and *Plasmodium* species, making it difficult to identify children with *P. falciparum*. The third study reported clinical data collected from children recruited to the SEQUAMAT intervention trial [78]. Because patients were stratified in 10-year age groups, it was not possible to extract important clinical data that supplemented those provided in the original report [20].

Of the studies included, only one systematically reported HIV status in children with severe malaria. Blood cultures were taken from at least some of the children with severe malaria in 7 studies [2], [12], [41], [47], [65], [79], but were routinely performed and reported in only one [2]. The proportions of deeply comatose children with BCS of 0, 1 and 2 were presented in 14, 13 and 13 studies, respectively, but the BCS-specific CFRs were only provided in 6, 3 and 3 studies, respectively.

### Heterogeneity in descriptive studies of severe malaria

There was substantial heterogeneity (*I* ^2^>80%) for all but three outcomes of interest. The exceptions were the proportions and CFR of children presenting with the overlap syndrome (*I* ^2^ 71.9 and 0%, respectively) and CFR for children presenting with a BCS of 0 (*I* ^2^ 0%). The overall heterogeneity (*I* ^2^), total estimated heterogeneity (τ^2^) and the effect of moderators on τ^2^ for the proportions of clinical features in descriptive studies are shown in Table 2. The same metrics for overall and feature-specific CFRs, and for BCS scores, are shown in Tables 3 and 4, respectively. Funnel plots for each outcome of interest did not indicate publication bias after the effects of moderators were incorporated into final mixed effects models (data not shown).

**Table 2:** Heterogeneity and the effect of moderators on heterogeneity for the proportions of children presenting with different clinical features in descriptive studies of severe *Plasmodium falciparum* malaria.

Outcome of interest	Number of studies with available data (n)	Overall heterogeneity from random effects model (*I* ^2^)	Total estimated heterogeneity in random effects model (τ^2^)	Effect of moderators on heterogeneity (τ^2^)				Final mixed effect model	
				Definition, τ^2^ (%)	Region, τ^2^ (%)	Year, τ^2^ (%)	Age, τ^2^ (%)	Moderators included	Residual heterogeneity, τ^2^ (%)
**Clinical manifestation proportion:**									
Deep coma	37	99.8%	0.0355	0.0177 (50)***	0.0318 (10)*	0.0343 (NS)	0.0292 (18)**	Def+Age	0.0167 (53)*
Metabolic acidosis	11	98.9%	0.0128	0.0070 (45)**	0.0131 (NS)	0.0081 (37)*	0.0139 (NS)	Def	0.0070 (45)**
Severe anemia	35	99.5%	0.0265	0.0228 (14)*	0.0266 (NS)	0.0258 (NS)	0.0273 (NS)	Def	0.0228 (14)*
Hypoglycemia	31	99.6%	0.0048	0.0046 (NS)	0.0043 (10)*	0.0049 (NS)	0.0049 (NS)	Region	0.0043 (10)*
Coma/anemia/acidosis overlap syndrome^a^	5	71.9%	0.0001	0.0001 (NS)	0.0001 (NS)	0.0001 (NS)	0.0001 (NS)	Nil	0.0001

NS, No statistically significant contribution to overall heterogeneity; Def, Definition; *, ** and ***, *P*-values<0.05, <0.01 and <0.0001, respectively;

a children with an overlapping syndrome comprising coma, severe anemia and any of: metabolic acidosis, respiratory distress or hyperlactatemia.

**Table 3:** Heterogeneity and the effect of moderators on heterogeneity for overall and clinical feature-specific case fatality rates in descriptive studies of severe *Plasmodium falciparum* malaria.

	Number of studies with available data (n)	Overall heterogeneity from random effects model (*I* ^2^)	Total estimated heterogeneity in random effects model (τ^2^)	Effect of moderators on heterogeneity (τ^2^)					Final mixed effect model	
Outcome of interest				Def, τ^2^ (%)	Region, τ^2^ (%)	Year, τ^2^ (%)	Age, τ^2^ (%)	Drug, τ^2^ (%)	Moderators included	Residual heterogeneity, τ^2^ (%)
**Case fatality rates:**										
Overall	45^a^	98.0%	0.0019	0.0017 (11)**	0.0016 (16)*	0.0020 (NS)	0.0019(NS)	0.0019 (NS)	Def+Region	0.0015 (21)*
Deep coma	50^b^	81.3%	0.0048	NA	0.0035 (27)**	0.0049 (NS)	0.0051 (NS)	0.0045 (NS)	Region	0.0045 (27)*
Metabolic acidosis	10	93.3%	0.0052	NA	0.0045 (NS)	0.0034 (35)*	0.0056 (NS)	0.0053 (NS)	Year	0.0034 (35)*
Severe anemia	23	95.8%	0.0035	NA	0.0026 (26)*	0.0034 (NS)	0.0038 (NS)	0.0034 (NS)	Region	0.0026 (27)*
Hypoglycemia	20	93.6%	0.0321	NA	0.0320 (NS)	0.0178 (45)***	0.0214 (33)**	0.0222 (31)*	Year+Age	0.0132 (59)*
Coma/anemia/acidosis overlap syndrome^c^	6	0.03%	0.0000	NA	NA	NA	NA	NA	NA	NA

NS, No statistically significant contribution to overall heterogeneity; Def, Definition; *, ** and ***, *P*-values<0.05, <0.01 and <0.0001, respectively.

a Includes 7 studies where overall case fatality rates are calculated according to treatment regimen.

b Includes 9 studies where case fatality rates due to deep coma are calculated separately according to treatment regimen.

c Children with overlapping syndrome comprising coma, severe anemia and any of: metabolic acidosis, respiratory distress or hyperlactatemia.

**Table 4:** Heterogeneity and the effect of moderators for proportions and case fatality rates according to Blantyre Coma Score in deeply comatose children from descriptive studies of severe *Plasmodium falciparum* malaria.

Outcome	Number of studies with available data (n)	Overall heterogeneity from random effects model (*I* ^2^)	Total estimated heterogeneity in random effects model (τ^2^)	Final mixed effect model		
				Region, τ^2^ (%)	Year, τ^2^ (%)	Age, τ^2^ (%)
**BCS Score in deep coma:**						
0	14	99.88%	0.0106	0.0118 (NS)	0.0060 (44)**	0.0087 (NS)
1	13	87.72%	0.0120	0.0132 (NS)	0.0113 (NS)	0.0117 (NS)
2	13	96.63%	0.0522	0.0568 (NS)	0.0354 (32)*	0.0554 (NS)
**CFR for BCS Scores**						
0	6	0%	0	NA	NA	NA
1	3	91.12%	0.0403	0.0283 (NS)	0.0601 (NS)	NA
2	3	61.18%	0.0015	0 (100)*	0 (100)*	NA

BCS, Blantyre Coma Score; CFR, Case Fatality Rate; NS, No statistically significant contribution to overall heterogeneity; * and **, *P*-values<0.05 and <0.01, respectively.

The definition of severe malaria used in each of the studies had a significant impact on heterogeneity for the proportions of deep coma, metabolic acidosis and severe anemia and hypoglycemia, accounting for 50%, 45% and 14% of total heterogeneity, respectively.

Geographic region was a significant moderator in the proportion of deep coma and hypoglycemia, accounting for 10% of heterogeneity in each. For the proportion with deep coma, region was not included in the most parsimonious mixed effects model that had definition of severe malaria and average patient age as the only significant moderators. When compared with African children, the proportion of children presenting with hypoglycemia was significantly lower in PNG children (10% [CI95 7–13%] versus 1% [0–3%], *P*<0.05; see Table 5).

**Table 5:** Geographic differences in the clinical features and outcome for severe malaria (data are proportions [%] with 95% confidence intervals [CI95] from random effects models).

Outcome of interest	Africa % (CI95)	Asia % (CI95)	Melanesia (Papua New Guinea) % (CI95)	Statistical significance in final mixed effects model
**Clinical feature proportion:**				
Deep coma	22 (16–29)	41 (16–65)	10 (3–16)	NS
BCS = 0*	14 (6–21)	-	13 (8–17)	NS
BCS = 1*	26 (19–34)	-	25 (14–37)	NS
BCS = 2*	57 (42–72)	-	61 (52–71)	NS
Metabolic acidosis	24 (17–31)	NA	17 (6–29)	NS
Severe anemia	32 (27–38)	40 (19–60)	23 (20–25)	NS
Hypoglycemia	10 (7–13)	7 (2–13)	1 (0–3)	<0.05
**Case fatality rate:**				
Overall	7 (6–9)	10 (8–13)	2 (0–4)	<0.05
Deep coma	19 (16–21)	19 (12–25)	8 (0–17)	<0.01
BCS = 0¶	39 (22–56)	-	37 (24–51)	NS
BCS = 1¶	28 (2–54)	-	0 (0–4)	NS
BCS = 2¶	7 (1–13)	-	0 (0–1)	<0.05
Metabolic acidosis	19 (16–21)	NA	8 (2–17)	NS
Severe anemia	8 (5–10)	27 (1–52)	1 (0–3)	<0.05
Hypoglycemia	27 (18–37)	42 (19–64)	14 (0–44)	NS

CI95, 95% confidence intervals; NS, Not statistically significant contributor to final mixed effect model.

* As proportion of children with deep coma.

¶ Case fatality rate for each BCS (Blantyre Coma Score).

**Figure 2:**
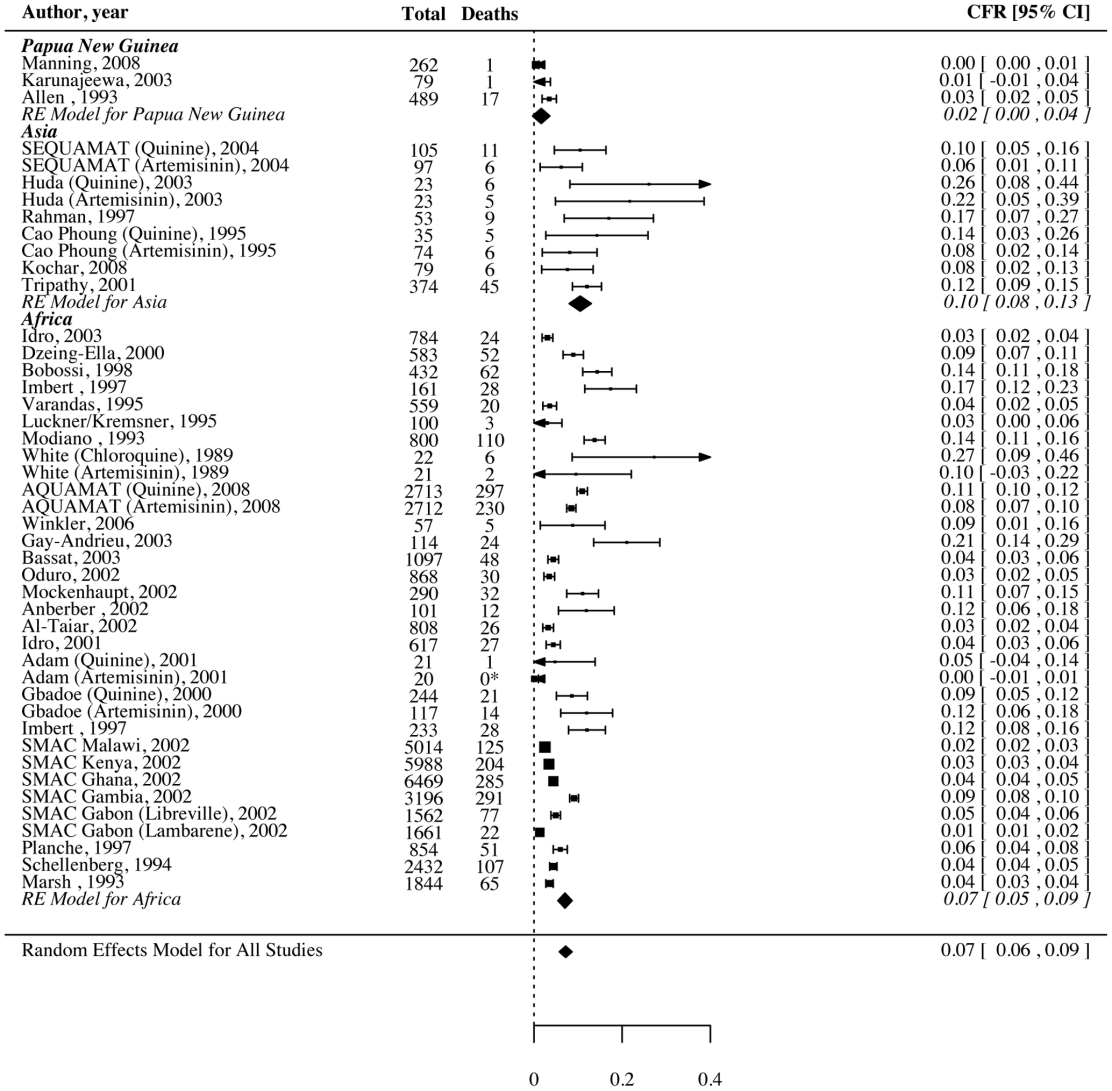
Forest plot showing random effects model of overall mortality due to severe malaria according to region.

Forty-five studies reported overall CFRs. A forest plot showing random effects models for overall mortality according to region is shown in Figure 2. The definition of severe malaria and region were significant moderators of heterogeneity, accounting for 11% and 16%, respectively. The final mixed effects model contained both variables. After adjustment for the definition of severe malaria used, the overall CFR was lower in PNG children when compared with African children (2% [0–4%] versus 7% [6–9%], *P*<0.05; see Table 5). Geographic region was also a significant moderator of heterogeneity in CFRs due to deep coma and severe anemia, accounting for 27% and 26% of total heterogeneity, respectively. PNG children had a significantly lower mortality due to cerebral malaria than African children (8% [0–17%] versus 19% [16–21%], *P*<0.05), while Asian children also had a higher mortality due to severe anemia than African children.

The average patient age in studies included in the meta-analysis was a significant moderator and was incorporated into the final mixed effects model for proportion of deep coma in severe malaria (18% of total heterogeneity) and mortality due hypoglycemia (33% of total heterogeneity). Artemisinin use was not a variable in any final mixed effect model for any outcome of interest.

For children with deep coma, the proportions in BCS categories were highly heterogeneous, with at least some of the heterogeneity accounted for by the year the study was performed. There were no apparent regional differences in the proportions of children with BCS of 0, 1 or 2, although no Asian studies were represented. The CFRs for children with a BCS of 0 had no heterogeneity (Table 4; CFR 38% [27–49%]). As a consequence, there were no regional differences observed. However, although limited to an analysis of 3 studies only, PNG children with a BCS of 2 had a lower mortality than African children (Table 5; 0% [0–4%] versus 7% [1–13%], *P*<0.05).

### Temporal trends in severe malaria mortality rates

**Figure 3:**
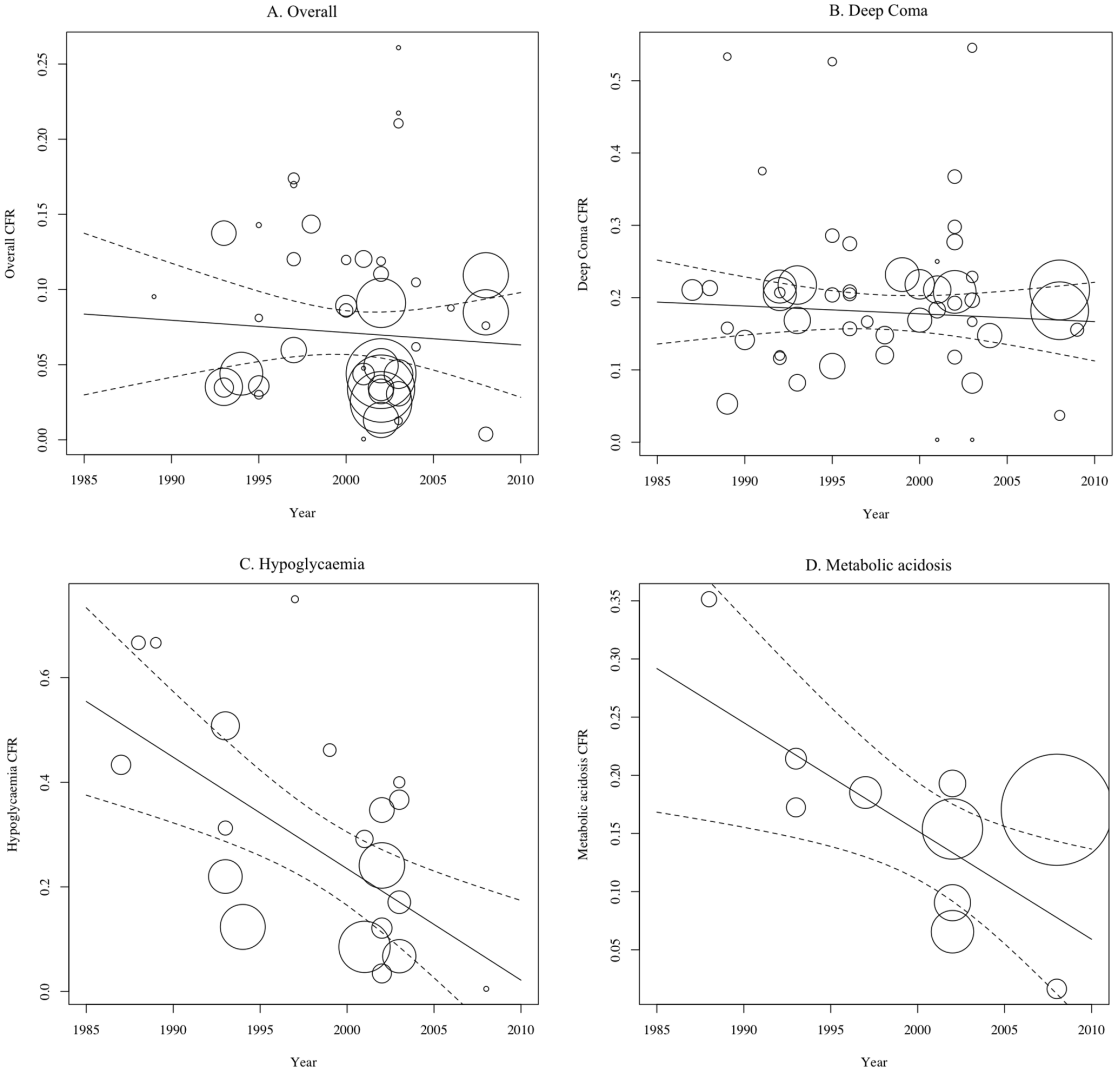
Temporal trends in overall case fatality rates (CFR) due to severe malaria (figure 3A) deep coma (figure 3B), hypoglycemia (figure 3C) and metabolic acidosis (figure 3D) (dashed lines represent 95% confidence intervals of predicted mortality rates).

There was no significant temporal trend in overall CFR (Figure 3A). The year in which the study was done was a significant moderator and contributed significantly to heterogeneity for hypoglycemia- and metabolic acidosis- associated CFRs, accounting for 35% and 45% of total heterogeneity, respectively (Figures 3C and 3D, respectively). The meta-regression line demonstrates declining CFRs due to hypoglycemia and metabolic acidosis. This is in contrast to the CFR due to deep coma that has remained constant over the past 30 years (Figure 3B).

## Discussion

The present systematic review and meta-analysis of descriptive and interventional studies of severe childhood *P. falciparum* malaria demonstrates that there are significant regional differences in the proportions of clinical features and outcome. PNG children present less frequently with hypoglycemia than African children, and they have lower CFRs overall and due to cerebral malaria. Asian children have a higher CFR associated with severe anemia than African children. The present analyses also show that, in contrast to population studies of malaria-attributable mortality [10], there has been no reduction in overall CFR or cerebral malaria-associated CFR over time despite declining CFRs due to both hypoglycemia and metabolic acidosis.

There was substantial overall heterogeneity between studies for nearly all outcomes that was only partly accounted for by moderator covariates. The definition of severe malaria used in each study had a large effect on heterogeneity for overall CFR and the relative proportions of deep coma, severe anemia and metabolic acidosis. When compared to ‘all malaria admissions’, the WHO 1990 definition had increased rates of deep coma, while metabolic acidosis and severe anemia were more frequent under the WHO 1990 and WHO 2000 definitions. This finding reflects the fact that the WHO 2000 definition and ‘all admissions’ have least restrictive inclusion criteria. The inclusion of prostration, impaired consciousness and at least two seizures in the WHO 2000 definitions are relevant examples.

Regional differences accounted for at least some of the overall heterogeneity between studies. When compared with their African counterparts, PNG children (in the only representative studies from Melanesia or Oceania) had lower overall and cerebral malaria-specific CFRs. The proportions of children from PNG with deep coma who had a BCS of 0, 1 or 2 were comparable to those in African studies. The CFRs for a BCS of 0 were extremely high but without heterogeneity. For children with BCS of 2, the CFR was lower in PNG children. These findings reflect epidemiological studies, historical information [80] and other data collected from Vanuatu and elsewhere in Melanesia in the 1980s and 1990s [8], [9]. Even though a Melanesian survival advantage is not universally endorsed [81], our analyses are the most robust to date and may reflect factors such as widespread availability of effective antimalarial therapy, immunological cross-protection from exposure to *P. vivax*, and a high prevalence of protective genetic polymorphisms such as South Asian Ovalocytosis (SAO) [82]–[84].

Locally prevalent protective genetic polymorphisms are, however, unlikely to account for the observed differences in CFRs. First, SAO prevalence is highly heterogeneous and been identified in coastal PNG peoples with Austronesian language lineage, but not elsewhere in Melanesia [85]. Second, and more importantly, the selection pressure maintaining this polymorphism in the population is protection against incident uncomplicated infection and possibly severe disease due to *P. vivax* rather than a survival benefit mediated via protection against cerebral malaria due to *P. falciparum* [86]. Other undiscovered novel local genetic polymorphisms may underlie the low Melanesian CFRs but, despite advanced bio-informatic and high-throughput molecular technologies, may be hard to identify because of the low number of outcomes and the consequent need for very large sample sizes.

Cross-sectional and longitudinal surveys in Melanesia have shown negative correlations between the prevalence of different *Plasmodium* species [82], [87], and mixed-species infections tend to be substantially less common in symptomatic compared to asymptomatic patients [87]. Epidemiologic evidence from Thailand has also shown that the incidence of severe malarial illness, but not mortality, is lower in patients with *P. falciparum* when there is a mixed infection with *P. vivax* [88]. In addition, *P. vivax* co-infection appears to abrogate the nadir in hemoglobin after treatment for falciparum malaria [89]. These observations have been interpreted as evidence that *P. vivax* protects against *P. falciparum* clinical disease [9], [88]. However, recent clinical data showing more severe clinical features and a substantially higher CFR in children with evidence of mixed *P. falciparum/P.vivax* infections in PNG is inconsistent with this concept [2].

Two alternative explanations for the low CFRs in Melanesian children with severe malaria include the low rates of hypoglycemia in this setting, as demonstrated in the present meta-analysis, and a more recent observation that concomitant bacteremia, particularly non-typhoidal salmonellae (NTS), occurs much less frequently than in African children with severe malaria. A very low prevalence of hypoglycemia in Melanesian children with severe malaria (1%) might account for lower CFRs observed in comatose children as well as overall CFR. Our analyses suggest that an absolute difference in hypoglycemia between PNG and other children of 8% might account for only 2% of the differences in overall CFR. Differences in nutritional status and hepatic glycogen reserves, pre-referral treatment with quinine with attendant hyperinsulinemia and as yet undiscovered genetic polymorphisms in glucose metabolism are possible explanations for geographical variability in hypoglycemia rates.

Concomitant bacteremia in severe childhood malaria in African children is well described, occurring in 5–16% of children [90]–[96], and is an independent risk factor for mortality with associated CFRs of up to 33%. NTS are an important cause of concomitant bacteremia in Africa, accounting for 7–58% of bacterial isolates and reflecting malaria-associated down-regulation of humoral and cellular immunity and/or disruption of the gut mucosa by microvascular sequestration [97]. Variability in the proportions of concomitant bacteremia and causative organisms between studies could be due to differences in local bacterial epidemiology (such as *S. aureus* nasal carriage rates or circulating *S. pneumoniae* serotypes), HIV seroprevalence and sickle cell disease rates, pre-treatment with antibiotics, and nutrition status [97]. Due to logistic challenges and cost in resource-poor settings, few studies have performed blood cultures systematically in children with severe malaria. Indeed, of the 65 studies in the meta-analysis, only 7 reported that blood culture were performed, usually as deemed clinically necessary rather than as a routine investigation. Although limited to a single study, a striking absence of concomitant bacteremia in admission blood cultures in children with severe malaria was observed recently in PNG [2]. In addition, no NTS were isolated from blood cultures from other children presenting with severe non-malarial illness [2]. This suggests that, in contrast to Africa where culture-positive NTS can be found in up to 4% of healthy children [98], these pathogens are uncommon in PNG and perhaps other parts of Melanesia. HIV prevalence in malaria endemic areas could also confound the results of the present study. However, standardised testing for HIV was performed in only one study and further analysis was not possible as a result.

There were no significant temporal trends for overall CFR or for CFRs due to deep coma and severe anemia in the present study. In addition, artemisinin use was not a significant moderator of any outcome of interest. These observations appear surprising in the light of recent models demonstrating that estimates of global malaria-attributable mortality have declined since 2004 [10], and the publication of the AQUAMAT [19] and SEQUAMAT [20] studies that showed a mortality benefit for artesunate over quinine in Asian and African children with severe malaria. However, models showing declining global malaria mortality do not incorporate inpatient data despite the fact that a significant proportion of children dying in tropical countries present to a health facility during their final illness [99]. Mortality estimates based on VA and VR may have limited validity in malaria-endemic countries [100], [101], while declining malaria-attributable mortality in these models are likely to reflect the success of malaria control programs at a population level rather than better in-patient care.

The lack of change in CFR due to cerebral malaria over recent decades could reflect diagnostic uncertainty, with a true decline masked by misdiagnosis of other causes of encephalopathic illness as cerebral malaria [102]. Alternatively, a significant proportion of children may present late in their illness with complications that are unresponsive to optimal resuscitation, and antimalarial and supportive therapy. In the present study, the lack of heterogeneity for, and magnitude of, the CFR for children presenting with a combination of cerebral malaria, severe anemia and evidence of metabolic acidosis highlights this point. A lack of heterogeneity was also observed for CFR in children with a BCS of 0. Regardless of geographic region or other moderators, the CFR for these specific groups of moribund children were 32% and 38%, respectively. On a more positive note, our data demonstrate significant improving temporal trends for CFRs due to metabolic acidosis and hypoglycemia. This is likely to reflect incorporation of routine use of intravenous 10% w/v dextrose and judicious use of fluid resuscitation [103].

Artemisinin use was not a significant moderator of any outcome of interest in the present analyses. Only one third of the studies involved an artemisinin derivative, nearly all of which were comparative intervention trials. The artemisinin formulations used varied widely in terms of derivative (artemether, artesunate or artemether) and route of administration (intramuscular, intravenous or rectal). Due to the limited number of studies and the different treatment regimens, we pooled the data for all preparations and routes of administration. This may have masked a beneficial effect of intravenous artesunate that was clearly demonstrated in AQUAMAT [19] and SEQUAMAT [20]. Despite this possible limitation, it is important to note that, even in a research setting, the overall mortality for African children treated with parenteral artesunate was still 8.5% and the CFR for deep coma was 18% [19]. Although both lower than observed for quinine treatment, these CFRs are consistent with the temporally stable respective mean estimates shown in Figure 3.

Our study had other limitations. First, the four Melanesian studies eligible for inclusion were from the same regional hospital in PNG. Each study was independent and conducted at different times, but the applicability of the present findings to other Melanesian geographical areas is unknown. Second, because of methodological issues described in the Results section above, two studies of severe malaria in Melanesian children were excluded from the analysis [76], [77]. From the tabulated data provided by Tjitra *et al.* [77], severe anemia and coma occurred in 20% and 1.7%, respectively, of all children admitted with malaria. Although these percentages are inaccurate because children with more than one severe manifestation were not counted, the rate of severe anemia is consistent with other Melanesian studies. By contrast, the percentage with deep coma appears lower than our data but may have been up to ∼3% if those children with more than one clinical manifestation had been included. The overall CFR for patients across all age groups with severe falciparum malaria was 2.2%, lower than in studies of patients from an Asian ethnic background. After adjustment for other variables, Asian ethnicity did not remain an independent risk factor for mortality, but the mortality trends for a predominantly Melanesian population in the Tjitra *et al.* study [77] accord with the low mortality observed in our meta-analysis. In the study published by Genton *et al.* [76], the limited ascertainment of clinical features and absence of CFRs precluded its incorporation in the present analysis. It is important to note that, in both excluded studies, blood glucose concentrations were not recorded. Therefore, neither would have influenced the striking regional differences observed in rates of hypoglycemia. A final possible limitation is that there were no studies from South America and, although we included studies in French, we did not search the Portuguese or Spanish literature. This may limit the generalizability of our results beyond Africa, Asia and Oceania, but it is unlikely that the omission of non-English, non-French literature would have impacted on our main findings [104].

In conclusion, the present meta-analysis supports the contention from individual studies and observations that there is a lower CFR for PNG children when compared with African children presenting with severe malaria. Although this may be mediated in part by low rates of hypoglycemia and concomitant bacteremia, there is still substantial heterogeneity across all studies. Although there is good evidence that CFRs due to hypoglycemia and metabolic acidosis have been declining, the lack of temporal change in overall and cerebral malaria CFRs in an era of improving access to parenteral artesunate should be a catalyst for renewed efforts to refine the diagnosis of severe malaria, improve pre-referral practices, optimise inpatient management and continue the search for effective adjunctive therapies.

## Supporting Information

Checklist S1 The supporting PRISMA checklist.
(DOC)
